# Artificial Insemination as an Alternative Transmission Route for African Swine Fever Virus

**DOI:** 10.3390/pathogens11121539

**Published:** 2022-12-14

**Authors:** Virginia Friedrichs, Darwin Reicks, Tobias Hasenfuß, Elisabeth Gerstenkorn, Jeffrey J. Zimmerman, Eric A. Nelson, Tessa Carrau, Paul Deutschmann, Julia Sehl-Ewert, Hanna Roszyk, Martin Beer, Jane Christopher-Hennings, Sandra Blome

**Affiliations:** 1 Institute of Diagnostic Virology, Friedrich-Loeffler-Institut, Südufer 10, 17493 Greifswald-Insel Riems, Germany; 2 Reicks Veterinary Research and Consulting, Saint Peter, MN 56082, USA; 3 Bundes Hybrid Zucht Programm, 21368 Dahlenburg-Ellringen, Germany; 4 Department of Veterinary Diagnostic and Production Animal Medicine, Iowa State University, Ames, IA 50011, USA; 5 Animal Disease Research and Diagnostic Laboratory, Department of Veterinary and Biomedical Sciences, South Dakota State University, Brookings, SD 57007, USA

**Keywords:** African swine fever virus, early virus detection, artificial insemination, pathogenesis, transmission, boar semen, vertical transmission

## Abstract

The rapid spread of the African swine fever virus (ASFV), causing severe disease with often high fatality rates in Eurasian suids, prevails as a threat for pig populations and dependent industries worldwide. Although advancing scientific progress continually enhances our understanding of ASFV pathogenesis, alternative transmission routes for ASFV have yet to be assessed. Here, we demonstrate that ASFV can efficiently be transferred from infected boars to naïve recipient gilts through artificial insemination (AI). In modern pig production, semen from boar studs often supplies many sow herds. Thus, the infection of a boar stud presents the risk of rapidly and widely distributing ASFV within or between countries. Daily blood and semen collection from four boars after intramuscular inoculation with ASFV strain ‘Estonia 2014’ resulted in the detection of ASFV genomes in the semen as early as 2 dpi, in blood at 1 dpi while semen quality remained largely unaffected. Ultimately, after insemination with extended semen, 7 of 14 gilts were ASFV positive by 7 days post insemination, and all gilts were ASFV positive by 35 days post insemination. Twelve out of 13 pregnant gilts aborted or resorbed at the onset of fever. A proportion of fetuses originating from the remaining gilt showed both abnormalities and replication of ASFV in fetal tissues. Thus, we present evidence for the efficient transmission of ASFV to gilts via AI and also to implanted embryos. These results underline the critical role that boar semen could play in ASFV transmission.

## 1. Introduction

As the etiological agent of African swine fever (ASF), a notifiable disease resulting in high fatality rates in Eurasian suids, the ASFV pandemic remains a threat to global pig populations and economies [1]. Originally, ASFV was endemic in sub-Saharan Africa, where it circulated in warthog and soft tick (genus: *Ornithodoros*) populations [2]. However, ASFV was introduced in Georgia in 2007; an event that initiated the pandemic spread of ASFV over the last 15 years. Numerous studies on the effects of socio-economic factors on ASFV spread were conducted in Sardinia [3]–a country on the brink of ASF eradication after decades of fighting the disease, with the last registered case in 2018 [4]. Despite progress in understanding and controlling the disease, the ongoing spread emphasizes the necessity of expanding our knowledge of drivers of disease dynamics, such as alternative transmission routes.

In this study, we investigated venereal transmission of ASFV from infected boars to female recipients. The contemporary pig industry relies on AI to optimize reproduction and produce high-quality progeny [5]. The semen used in AI often originates from boar studs, distributing 3 billion spermatozoa/dose/sow from high-health boars selected for their genetic potential [6]. This enables the insemination of many females without the need for each individual farm to purchase, house, and feed their own boars. Notably, more than 90% of all breeding sows are inseminated artificially in many countries [7], which emphasizes the potential for the venereal transmission of ASFV. At boar studs, semen is collected and distributed, often nationwide or across borders, via same-day or next-day delivery. Since boar semen can act as an efficient transmission agent for a variety of viral diseases, e.g., pseudorabies virus (PRV [8]), foot-and-mouth disease virus (FMDV [9]), porcine reproductive and respiratory syndrome virus (PRRSV [10]), swine vesicular disease virus (SVDV [9]), porcine parvovirus (PPV [11]), porcine picornaviruses [9], and possibly ASFV, comprehensive insight into the involvement of boar semen in ASFV transmission is essential. This route of transmission not only presents a risk to commercial pig farming but also small (backyard) farms. In many countries (e.g., Serbia, Romania, India, and most countries of south-east Asia), the majority of agricultural producers rely on backyard farming, with a few boars relied upon to produce the majority of piglets in villages in close proximity — putting the livelihood of many families at risk [12].

Although data regarding the role of the porcine male reproductive tract on ASFV transmission is scarce, Thacker et al. (1984) mentioned the potential risk of ASFV transmission from boars to sows through semen [9] and advanced the theory of AI as an efficient transmission route. Recent studies by Roszyk et al. (2021) supported this hypothesis by confirming the presence of both viral genome and infectious virions in the gonadal tissue of swine infected with ASFV strains of various genotypes (gt). In these studies, the ASFV genome and infectious virions were detected in testes from sexually mature domestic pigs inoculated with Zambian ASFV isolates ‘KAB 6/2’ (gt XI) and ‘SUM 14/11’ (gt XIII) [13]. Studies with adolescent wild boar produced similar results for ASFV strains ‘Belgium 2018/1’ (gt II) and ‘Germany 2020’ (gt II) [13,14].

Based on these findings, we performed a study to specifically address the question of ASFV transmission from boars to naïve females via AI. To this end, we inoculated four boars with ASFV isolate ‘Estonia 2014’ (gtII: [15]) and used pooled, extended semen to perform AI on 14 gilts. The assessment of the ASFV genome and infectious virions in the reproductive organs of boars and gilts allowed for comprehensive insight into a largely unexplored area of ASFV pathogenesis. We not only present evidence for efficient transmission of ASFV to gilts via AI, but also to implanted embryos. To meet the need for non-invasive ASFV surveillance sampling in boar studs, the suitability of other matrices (oral fluid, serum, fecal samples) for early ASFV detection was assessed on a limited scale.

## 2. Materials and Methods

### 2.1. Experimental Design

The trial involved four adult breeding boars: two Large White boars (493/494 days of age at start of the trial) and two Pietrain boars (396 days old). Furthermore, 14 gilts (Large White lineage ‘Viktoria’, 240 days old, originating from 12 litters) and two additional cross-bred indicator boars of the same age were included in the trial at a later time point. All animals were kept in high-containment facilities at the Friedrich-Loeffler-Institut (FLI), Isle of Riems, Germany. Prior to the transfer to the FLI, all animals tested negative for ASFV via qPCR. An acclimation period of roughly one week was provided to all animals. The animals originated from a commercial pig breeding company (Bundes Hybrid Zucht Programm, BHZP, Dahlenburg-Ellringen, Germany) to ensure the acquisition of well-performing breeding boars and synchronized gilts of high hygiene status. Synchronization of gilts was performed at the holding of origin using altrenogest (Regumate^®^, MSD Animal Health, Rahway, NJ, USA) over a period of 18 days. Estrus induction was facilitated by administration of cloprostenol (Estrumate^®^, MSD Animal Health, Rahway, NJ, USA) on the day of transport and pregnant mare serum gonadotropin (Pregmagon^®^, Ceva, Düsseldorf, Germany) at the FLI one day later.

The animal experiment was performed in accordance with the current German Animal Welfare regulations and approved by the responsible authority (Landesamt für Landwirtschaft, Lebensmittelsicherheit und Fischerei Mecklenburg-Vorpommern [LALLF M-V]) under file reference 7221.3-1-071/21.

All animals received a unique ear tag to enable definite identification of individuals: #1 and #2 (Large White Boars), #3 and #4 (Pietrain Boars), and #530, #533, #534, #536, #539, #567, #576, #610, #614, #627, #630, #635, #646, #654 (gilts). The boars were divided into two stable units, where they were kept individually in pens but within close proximity. This allowed species-appropriate social behavior in accordance with the German Ordinance on the Protection of Animals Kept for Farming Purposes without risk of injury. Gilts were divided randomly into groups of seven due to pen size restriction, and each group was kept in a separate stable unit. All individuals in the trial were kept under similar housing conditions. Estrus-indicator boars were divided between gilt groups. Of note, these boars were also kept individually, but were able to interact with gilts through fencing.

The initial study design used oro-nasal inoculation of boars to mimic natural conditions without vector involvement. To this end, the breeding boars (*n* = 4) were inoculated oro-nasally with 10 mL of a spleen suspension containing approximately 1 × 10^5^ hemadsorbing units 50% (HAU_50_) per mL of the ASFV strain ‘Estonia 2014’ (gt II). This virus strain had been shown to be an attenuated phenotype in previous studies [15] and was chosen to allow long-term follow-up of the boars. This initial design was modified upon the absence of the ASFV genome in all boars at 4 dpi, and intramuscular inoculation (IM) was performed with approximately 1 × 10^4^ HAU_50_ of the same ASFV strain. This modification was driven by the need to ensure active viraemia in boars within the timeframe of gilt synchronization (the modification was approved by the animal welfare authority).

After IM inoculation, semen was collected at close intervals (see Section 2.3) using a three gloved method: (first glove) the prepuce was evacuated, (second glove) the penis was brought to full extension and dried with a paper towel, and (third glove) the boar was collected with the tip of the penis positioned so as to deliver semen directly into the collection container. Semen was analyzed for basic quality parameters and the ASFV genome. These results determined the AI semen donors (see below).

Prior to AI, the boar semen was diluted (1:5) with a BTS semen extender (Minitube, Tiefenbach, Germany). The semen diluent was pre-warmed to 37 °C to ensure the viability of spermatozoa. To conduct AI, the diluted semen from all boars (except #4) was pooled in equal parts and insemination tubes (Minitube) were prepared to contain 3 billion spermatozoa in 60 mL. Most gilts (10 out of 14) were inseminated twice over two days, i.e., while they showed tolerance reflex, using boar semen collected at days 4 and 5 post-IM inoculation. All gilts were inseminated with the same batch of semen. Four gilts (#536, #610, #627, #630) were only inseminated once due to the absence of estrus signs on day 2 of AI. To minimize reflux of semen and subsequent contamination of the pen with ASFV-contaminated semen, super plus-sized tampons (O.B. tampons super plus, Johnson & Johnson GmbH, New Brunswick, NJ, USA) were used (3 tampons/gilt with ~20 mL holding capacity each), in combination with post-insemination disinfection of the vulvar area and pen (MENNO^®^ Neopredinol animal wash, anifarm, Uelzen, Germany).

Throughout the study, animal well-being was assessed daily using a comprehensive clinical scoring (CS) system designed to monitor changes in animal behavior and appearance linked to disease progression; e.g., skin and gait alterations or reduced feed intake ([16], modified). Animals were euthanized either upon reaching a clinical score of 15 points (humane endpoint, [17]) or upon the development of clinical signs that were classified as intolerable. Furthermore, rectal temperatures were taken daily, with a rectal temperature of ≥40.0 °C recorded as fever.

Euthanasia was conducted by exsanguination after inducing deep anesthesia with a combination of 2.2 mg/kg tiletamine/zolazepam (Zoletil^®^, Virbac, Bad Oldesloe, Germany), 4.4 mg/kg xylazine (Xylavet 2%, Medistar, Ascheberg, Germany), and 2.2 mg/kg ketamine (Ketamin 10%, cp-pharma, Burgdorf, Germany). Where exsanguination was not feasible, euthanasia was done via injection of Tetracainhydrochloride/Mebezoniumiodide/Embutramide (T 61, msd-Tiergesundheit) intracardially after inducing deep anesthesia as above. Following exsanguination/euthanasia, all animals were subjected to a comprehensive necropsy, as described elsewhere [18,19].

### 2.2. Viruses and Cells

For oro-nasal inoculation of the boars, a porcine spleen from a previous trial on the pathogenesis of ‘Estonia 2014’ was pulverized using sterile sea sand and then titrated on macrophages derived from peripheral blood mononuclear cells (PBMCs), as described elsewhere [15].

PBMCs were isolated from EDTA blood from healthy donors kept in the FLI quarantine facility. Cells were extracted by mixing whole blood with a 10% Hanks dextran solution (Sigma-Aldrich, St. Louis, MO, USA) at a ratio of 1:10. After 90 min incubation, the PBMC-containing supernatant was collected, erythrocytes diluted 1:10 with PBS, and then stored at 4 °C. Cells were washed and seeded at a density of 5 × 10^6^ cells/mL in Dulbecco′s Modified Eagle′s Medium (DMEM, supplemented with 10% fetal calf serum and 0.01% Penicillin/Streptomycin, Gibco) and incubated to perform hemadsorption tests (HATs), as described elsewhere [20]. For 96-well plates, 5 × 10^5^ cells/well were seeded; for a 24-well plate, 2.5 × 10^6^ cells/mL. All incubation steps were conducted at 37°C in a humidified atmosphere and in the presence of 5% CO_2_ for 24 h, unless stated otherwise. Recombinant colony-stimulating factor 2 (CSF2) was added at a concentration of 2 ng/mL after the first incubation.

The ASFV inoculum was thawed and titrated on differentiated macrophages in a 96-well plate. Erythrocytes were added to the cell medium at a ratio of 1:40 and rousette formation was evaluated after 24 and 48 h. Prior to IM inoculation, the virus stock was titrated on PBMC-derived macrophages. For oro-nasal inoculation, a titer of 1 × 10^5^ HAU_50_/mL in a total volume of 10 mL was used; for IM inoculation, 1 × 10^4^ HAU_50_ were administered in a volume of 1 mL. To ensure accuracy, the inoculum was back-titrated in triplicate.

### 2.3. Sample Collection and Processing

To assess ASFV kinetics in boar semen, semen was collected at days −7, −3, −2, −1, 0, 2, 3, 4, 5, 14, and 20 post-IM inoculation. Notably, at days 14 and 20 pi, semen was only obtained from boar #3. Although the initial trial design included daily collection after oro-nasal inoculation (see Section 2.1), semen collection was not done on day 1 post-inoculation to comply with animal welfare permissions and assure semen quality for AI.

For semen collection, each boar was provided a dummy for mounting. Semen was obtained following the principles of the three-glove free-catch semen collection recommended by Reicks et al. [21]. In this method, semen was collected with a clean glove grasping a pre-dried penis and the semen collected directly from the penis into the cup without any contact with the glove. Immediately thereafter, the cup was placed in a thermal mug (Minitube). To prevent bulbourethral secretions or other components from contaminating the semen, specialized collection bags with filters were used (Minitube).

To enable daily assessment of systemic genome loads, blood samples were collected from boars on days −7, −3, −2, −1, 0, 1, 2, 3, 4, 5, 14, and 20; however, at 1 dpi, blood samples were only obtained from boars #1 and #4. Blood samples (with and without EDTA) were obtained from each boar during semen collection through venipuncture of the saphenous vein or neighboring blood vessels using aspiration collection tubes (KABE Labortechnik, Nümbrecht, Germany).

Boar semen and blood samples were processed on the day of collection. Semen was diluted with the BTS semen extender, as described above, and kept at 37 °C to ensure spermatozoa viability. Spermatozoa counts and viability/motility were assessed for each collection. Furthermore, 500 µL of whole semen was centrifuged at 1000 rpm for 5 min to obtain the cell-rich fraction, and the pellet was collected in 100 µL PBS. Extraction of DNA from whole semen, the cell fraction, and blood samples allowed detection of the ASFV genome in all three matrices on the same day.

Oral fluid samples were collected from each boar after semen collection by providing access to 1.5 Check meaning retained 3-strand cotton rope for 30–45 min. Thereafter, the rope was cut to be placed in a 50 mL centrifugation tube with 0.5 mL tubes at the bottom, and centrifuged at 3000× *g* for 10 min at 4 °C. The same fluid collection procedure was used for dummy swabs. Collected fluids were stored at −80 °C. Additionally, feces (taken from rectum) and rectal swabs were collected from each boar on all semen collection days, and the collection dummy was swabbed (Swiffer) upon completion of semen collection on 3–5 dpi. Swabs were soaked in DMEM without supplements for 2 h and liquids were then stored at −80 °C.

Caudal vein blood samples were obtained from gilts on days 7, 14, 21, and 28 after AI. All blood samples were processed and ASFV genome loads were assessed on the respective day of sampling. Additionally, blood swab suspensions were tested for ASFV-specific antibodies.

The following samples were collected from all individuals (except indicator boars) at necropsy and subsequently tested for ASFV: blood, serum, tonsil, mandibular lymph node (mnLN), lung, kidney, spleen, liver, gastrohepatic lymph node (ghLN), inguinal lymph node (ingLN), and popliteal lymph node (pLN). Samples from the reproductive tracts of boars included testis, epididymis, prostate, vesicular glands, and bulbourethral glands. In gilts, tissues collected included vestibulum, vagina, cervix, uterus, salpinx, and ovaries. A portion of each organ was cut into pea-sized pieces and stored at −80 °C. One piece was placed in a 2 mL centrifugation tube with 1 mL PBS and a 5 mm metal bead, then subjected to qPCR after homogenization at 30 Hz for 3 min using a tissue lyzer (TissueLyzer II, Qiagen, Venlo, The Netherlands).

### 2.4. DNA Extraction and qPCR

To extract nucleic acids for downstream molecular tests, 100 µL of each tissue homogenate, blood obtained during necropsies, and serum were processed using the NucleoMag^®^ VET Kit (Macherey-Nagel, Düren, Germany) on a KingFisher 96 Flex System (Thermo Fisher Scientific, Waltham, MA, USA) according to the manufacturer’s instructions. Proven ASFV genome-negative serum was included as an extraction control. Since qPCR was conducted utilizing the VetMAX™ African Swine Fever Virus Detection Kit (Thermo Fisher Scientific), the internal control DNA was added to the extraction process to assess qPCR performance, as instructed by the manufacturer.

For daily qPCR testing, DNA from whole semen, semen cell fraction, or blood was extracted from 80 µL of each specimen using the QIAamp Viral RNA Mini kit (Qiagen). As described above, ASFV genome-negative serum was included as the extraction control. After extraction, qPCR was performed utilizing the virotype^®^ ASFV PCR kit (Indical, Leipzig, Germany). For quality assurance, the internal control provided by the manufacturer was included to define possible inhibition of qPCR reactions. All qPCR reactions were conducted on a C1000^TM^ thermal cycler, equipped with the CFX96^TM^ Real-Time System (Bio-Rad, Hercules, CA, USA). Data visualization was performed with GraphPad Prism 9 (GraphPad Software Inc., San Diego, CA, USA).

### 2.5. Virus Isolation

The hemadsorption tests (HAT) were performed to detect the presence of infectious virions in semen, blood, spleen, and tissues from boar and gilt reproductive tracts. Initially, PBMCs were seeded into a 24-well plate and macrophages were differentiated, as described above. Subsequently, 200 µL of tissue homogenate or 200 µL of diluted EDTA blood (1:10 in PBS) was added to differentiated macrophages in duplicate. After 72 h, the plate was frozen at −80 °C (≥ 24 h) to ensure throughout the complete freezing and lysis of cells. Thereafter, the supernatant was subjected to a HAT to assess the presence of infectious ASFV particles. Each technical replicate (*n* = 2) of the blind passage was assessed in technical replicates (*n* = 4) in HAT. The results were divided into negative (—, all wells negative), weak-positive (•, up to 4 wells positive), positive (••, 4–8 wells positive), and strong positive (•••, 4–8 wells positive, high rousette counts).

### 2.6. Serology

To assess the seroconversion of boars and gilts after inoculation, serum samples were routinely tested using several methods for detecting antibodies targeting ASFV antigens. This included all boar serum samples, blood swabs (in PBS) from gilts, and serum samples collected during necropsy. Samples were tested using two complementary ELISA kits: (I) ID Screen^®^ ASF Indirect (ID.vet, Montpellier, France), detecting antibodies targeting ASFV p32, p62, p72; and (II) Ingezim PPA COMPAC (Ingenasa, Madrid, Spain), detecting p72 targeting antibodies. Assays were performed according to the manufacturer’s instructions. All serum samples obtained from boars were subjected to these tests. Of gilts, blood swabs were soaked in PBS and served as matrix for serology. Final serum samples obtained during necropsy were collected from all animals and subjected to ELISA. Furthermore, all serum samples collected at necropsy and all ELISA-positive samples were tested on the immunoperoxidase test (IPT). Since the IPT is currently the most sensitive serological test to detect ASFV antibodies, the IPT results served as reference for the ELISA results.

## 3. Results

### 3.1. Clinical Signs, Clinical Scores (CS), and Macroscopic Lesions

None of the four boars oro-nasally inoculated with the attenuated ASFV strain ‘Estonia 2014’ (1 × 10^5^ HAD_50_/mL) developed fever or other clinical signs. Daily qPCR confirmed the absence of the ASFV genome in blood at 4 dpi. Given that the viral genome was detected by (or before) 4 days after oro-nasal inoculation in all previous studies [15,19,22], it was assumed that productive infection had not been established. This assumption was strengthened by the absence of ASFV-specific antibodies in both Large White boars and the late occurrence of antibodies in the Pietrain boars (later than 10 days post-IM inoculation).

**Figure 1:**
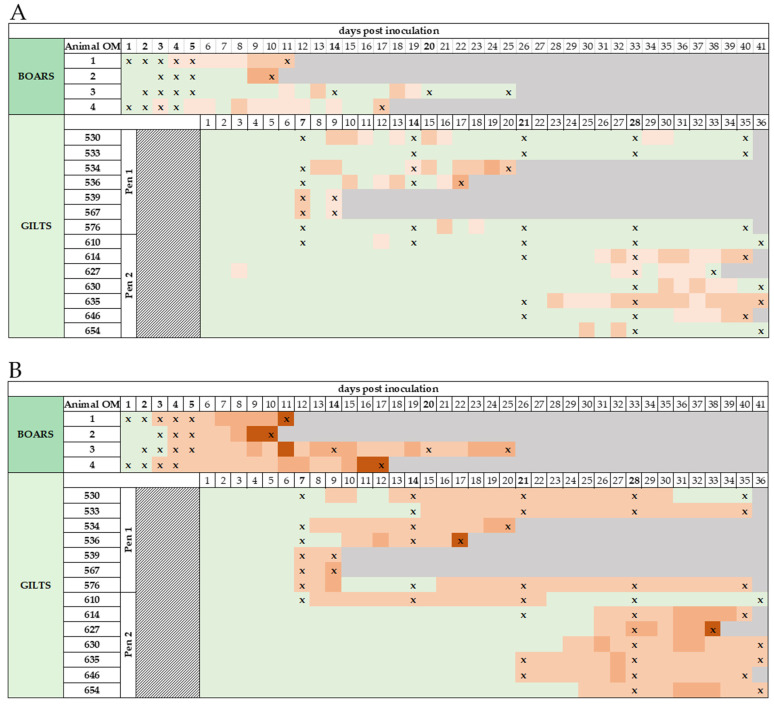
The rectal temperatures and clinical scores of boars and gilts were taken each day after inoculation/insemination. (**A**) Schematic summary of rectal temperatures after ASFV inoculation. Temperatures [°C] were color-coded as follows; green: ≤40, light orange: 40.1–40.4, orange: 40.5–41, dark orange: ≥41.1, grey: animal dead. (**B**) Schematic summary of clinical scoring points of each individual along the course of the trial. Scoring points were color-coded as follows; green: 0, light orange: 0.5–5, orange: 5.5–10, dark orange: 10.5–15, grey: animal dead. Symbol (X) marks days of positive qPCR results.

Following IM inoculation, all boars developed fever. Boar #4 showed elevated temperatures (40.1 °C) from 3 dpi, boar #1 (40.1 °C) and #2 (40 °C) at 4 dpi, and boar #3 at 6 dpi (Figure 1A). The two Large White boars (#1, #2) rapidly declined in health and showed severely reduced alertness, anorexia, and unwillingness to stand. The animals developed body temperatures of up to 41.1 °C (#2, 9 and 10 dpi), accompanied by shivering and discoloration of skin and eyes. Close to the humane endpoint, both boars presented abdominal cyanosis and hematomas. The prompt increase in body temperature and other clinical signs resulted in an increase of the CS (Figure 1B). While both boars had CS scores of 4 points on day 6 post-inoculation, boar #1 reached the humane endpoint at 11 dpi with pulmonary failure, epistaxis, and a CS of 15. Boar #2 reached the humane endpoint at 10 dpi, with 14.5 collective points.

Both Pietrain boars (#3, #4) also presented elevated temperatures up to 40.5 °C (#3 at 13 and 18 dpi, #4 at 8 and 17 dpi). However, both showed steady temperatures of ~40 °C on most days, occasionally below 40 °C. It is of note that the CS of boars #3 and #4 increased to 12 points (#3 at 11 dpi) at the peak of ASFV viraemia but declined starting at 12 dpi. Ultimately, both boars reached the humane endpoint due to secondary bacterial infections. Boar #4 was euthanized at 17 dpi due to a high-grade panaritium in the front right leg and initial signs of phlegmon. Boar #4’s display of aggressiveness and unwillingness to mount the dummy may be attributed to his discomfort. Boar #3 presented respiratory distress due to pulmonary infection and discoloration of the ears starting at 13 dpi, as well as increasing difficulties in standing. For two weeks, the CS of boar #3 ranged from 4–7 until euthanasia at day 25 pi. Macroscopic lesions during necropsy resembled ASFV infection (with bacterial complication in the Pietrain boars).

After AI, some gilts showed elevated temperatures and mild clinical signs, e.g., reduced feed intake and lethargy, at 7 dpi (#539, #567, #576). One gilt in pen 1 (#530–#576) started showing signs at 15 dpi (Figure 1B). In pen 2 (#610–#654), only one gilt showed early signs of disease at 8 dpi (#610), while all pen mates showed the first signs at 21 dpi (#635, #646), 24 dpi (#630), 25 dpi (#654), and 26 dpi (#614, #627). Notably, gilt #610 recovered from ASFV infection and presented with a CS of 0 starting at 28 dpi. Gilts #539 and #567 were euthanized on day 9 pi to obtain peak-viraemia samples, with a CS of 4 and 8.5, respectively. Gilt #536 reached the humane endpoint at day 17 pi due to severe rectal bleeding and pulmonary failure (CS 12). Gilt #534 presented considerable yellow discoloration of the skin, eyes, and mucous membranes on day 19 pi. The animal, although presenting a moderate clinical score of 8.5, had to be euthanized due to liver failure at day 20 pi. Gilt #627 started showing clinical signs at 26 dpi (CS 4), and its overall well-being rapidly declined until euthanasia at 33 dpi due to reaching the humane endpoint with 14.5 points. Furthermore, all gilts presented cyanosis/dermal petechiae of various severity during the trial. Based on uterus and ovary morphology analyzed during necropsies, all gilts with the exception of gilt #530 implanted embryos. From day 26 pi on, all gilts except gilt #533 aborted or resorbed, which correlated with temperature elevation (witnessed abortions where embryos/fetuses could be recovered: #627 at 26 days, #630 at 28 days, #654 at 26 days post-insemination). All aborted fetuses that could successfully be recovered were tested for ASFV. Necropsy of uteri in pregnant gilts revealed embryos/fetuses in distinct stages. For example, gilt #539, necropsied early after insemination at 9 dpi, presented (I) embryos of expected size and morphology, (II) embryos in various stages of resorption, and (III) remains of already resorbed embryos. Gilt #533, necropsied later after insemination at 35 dpi, showed (I) fetuses of expected size and morphology, (II) fetuses with red discolorations of skin/amniotic fluid or anomalies in size/morphology, and (III) cavities with empty amniotic sacs.

### 3.2. Early Detection of ASFV Genome in Various Matrices Obtained from Boars

#### 3.2.1. Boar Semen and EDTA Blood

**Figure 2:**
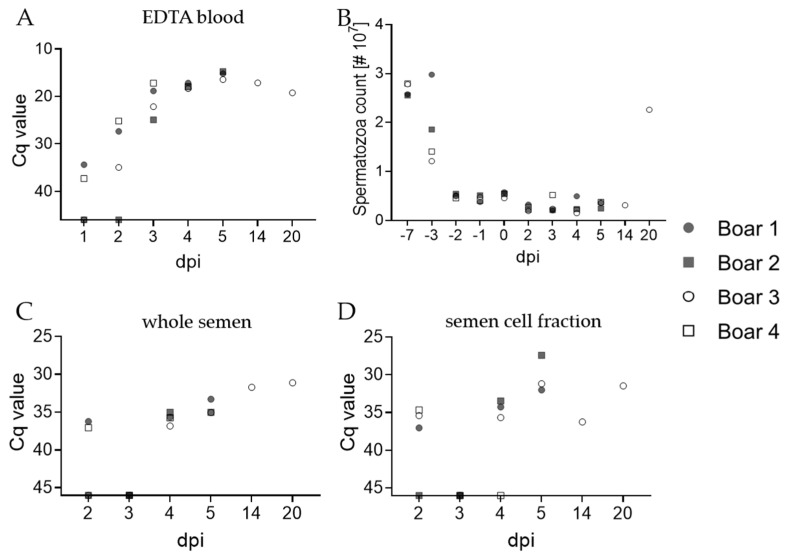
Kinetics of ASFV genomes in blood and semen of boars, as well as effects on spermatozoa count. Detection of the ASFV genome was conducted on blood (**A**) as the most sensitive matrix. (**B**) Spermatozoa of each boar were counted prior and post-inoculation with ASFV ‘Estonia 2014’. The amount of ASFV genome was further assessed in whole semen (**C**), and semen cell fractions (**D**) by qPCR. Results are depicted as Cq values. Each individual is represented by a symbol.

To assess the risk of transmitting ASFV via contaminated boar semen, we monitored the ASFV genome loads in semen daily from days 2 to 5 pi. Since blood was previously defined as the most suitable matrix for early ASFV detection [23], blood samples were included in daily sampling as a reference. Anticoagulated blood samples of boars #1 and #4 were ASFV positive as early as day 1 pi, with Cq values of 34.4 and 37.3, respectively (Figure 1 and Figure 2A). Boar #3 was positive in blood at day 2 pi with a Cq value of 35, while boar #2 was first positive at 3 dpi with a Cq value of 25. The Cq values of all boars increased until 5 dpi, with peak Cq values of 15.2, 14.7, and 16.4 for boars #1, #2, and #3, respectively. Due to aggressive behavior upon onset of clinical signs, no sample could be acquired from boar #4 on day 5 pi. A moderate increase in Cq values was visible starting at 14 dpi (#3), whereas the animal was still highly positive with a Cq value of 19.3 at 20 dpi.

Infection with ASFV and acute fever/lethargy had no effect on the spermatozoa count (Figure 2B) and motility, nor did we observe an increase in anomalous spermatozoa or decrease in boar’s motivation to mount (with the exception of boar #4) on sampling days prior to 14 dpi. In samples taken at 14 and 20 dpi (boar #3), abnormalities in morphology and reduced motility were observed. Results between whole semen samples and corresponding cell fractions differed in that boars #1 and #4 were positive in semen at 2 dpi with Cq values of 36.2 and 37, but boar #3 was positive on the same day upon consideration of the cell fraction results with a Cq value of 35.4 (Figure 2C,D). All boars were negative at 3 dpi, and all were positive at day 4 and 5 pi. The Cq values in whole semen and cell fractions slightly decreased over time, but never below 27.4 (#2, cell fraction at 5 dpi). Boar #3 remained positive in both whole semen and the corresponding cell fraction at sampling 14 and 20 dpi.

#### 3.2.2. Oral Fluids, Dummy Swabs, Serum, and Rectal Samples

**Figure 3:**
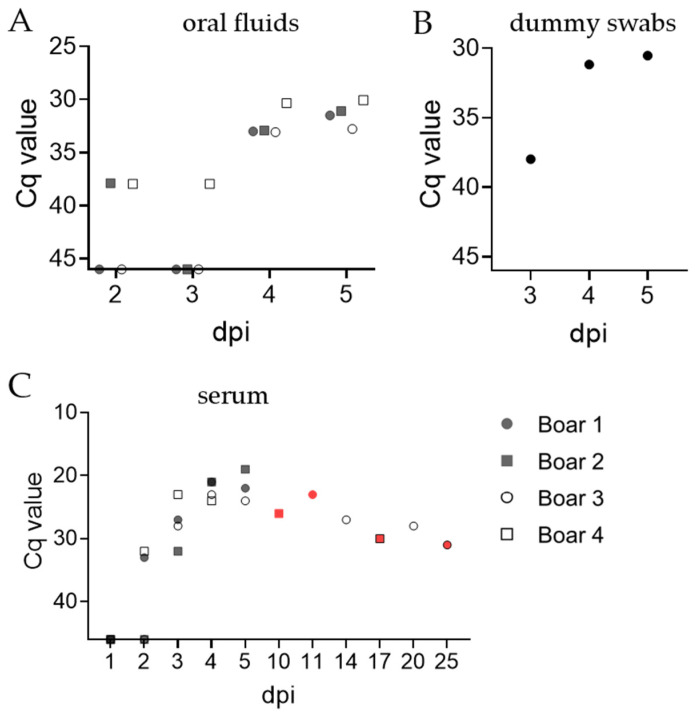
Detection of ASFV genome in cotton ropes (**A**), dummy swabs (**B**), and serum samples (**C**) of boars by qPCR. (**A,C**) Each symbol represents the mean of technical triplicates for each boar. (**B**) Each symbol represents the mean of technical triplicates per individual. Red symbols indicate samples obtained during necropsy.

Oral fluids were tested by qPCR to evaluate their suitability for early detection of ASFV infection. We detected the ASFV genome in oral fluid as early as 2 dpi at a Cq value of 38 for boar #2 and #4 (Figure 3A). However, only boar #4 was positive on day 3 dpi. All boars were positive in oral fluid on days 4 and 5 pi. A moderate increase in the ASFV genome was observed in samples from Large White boars (Cq between days varied by ±1.5 for boar #1, ±2 for boar #2), while Cq values of the Pietrain boars remained stable (33 for boar #3, 30 for boar #4). Dummy swabs collected at days 3, 4, and 5 were ASFV positive qPCR (Figure 3B). The amount of detectable ASFV genome in dummy swabs increased from day 3 to 4 pi (ΔCq 7), but remained stable from day 4 to 5 pi. Detection of genome in serum samples was comparable to corresponding EDTA blood samples (Figure 3C). In line with findings derived from blood samples at 1 dpi, boars #1 and #4 were the first to become ASFV positive, although not until day 2 pi. Notably, results obtained with serum samples were largely comparable with corresponding EDTA blood samples; however, Cq values were considerably higher in serum.

Rectal samples (feces and rectal swabs) resulted in detection of the ASFV genome later than all other matrices tested. Rectal swabs of boar #3 were positive on days 4, 14, and 20 pi, with high Cq values (Figure S1A). On the other hand, only boar #1 was positive in feces on days 4 and 5 pi, with Cq values of 36.7 and 34.6, while all other fecal samples remained negative by qPCR (Figure S1B).

### 3.3. ASFV Genome Load in Organs of Boars and Gilts

#### 3.3.1. Organ Samples Routinely Tested in ASFV Pathogenesis Studies

**Figure 4:**
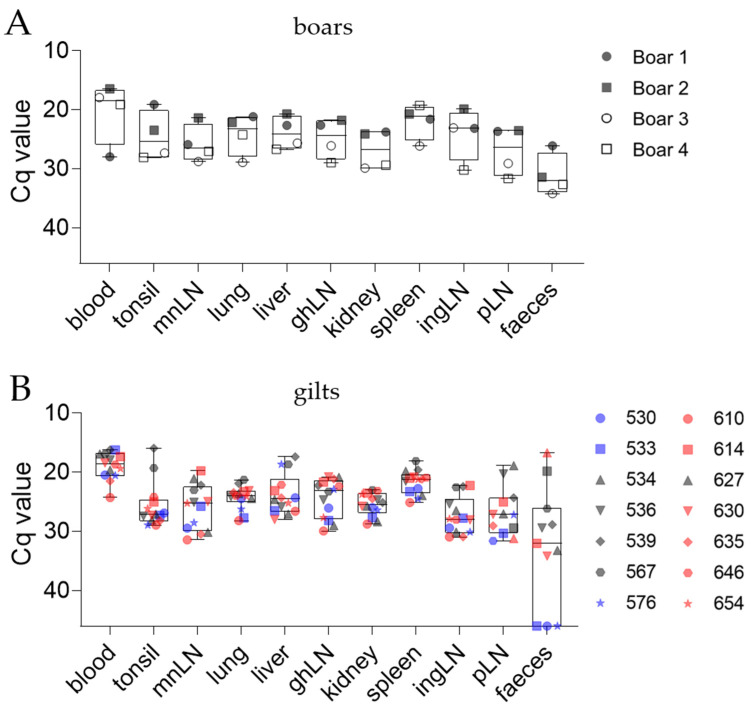
Detection of the ASFV genome in organ samples of boars (**A**) and gilts (**B**) by qPCR. Boxes represent 25/75 percentiles, including the group median with min. and max. values, each individual is represented by a symbol. Grey symbols represent animals that reached the humane endpoint and were euthanized prior to trial termination. Colored symbols represent individuals euthanized at the scheduled end of the trial; blue symbols represent pen 1, red symbols pen 2.

To gain insight into ASFV dissemination within infected boars and gilts, we analyzed ASFV genome loads in tonsil, mnLN, lung, spleen, kidney, liver, ghLN, ingLN, and pLN, as well as EDTA blood and fecal samples obtained during necropsy. Since all boars had to be euthanized prior to reaching the scheduled end of the trial (day 90 pi), the ASFV genome was detected in all organs tested (Figure 4A). Just as the severity of acute ASFV infection differed between Large White boars and Pietrain boars, differences were observed in Cq values of the ASFV genome in all organs, except blood. Subsequently, changes in Cq values between days were evaluated and defined as ΔCq. Variations among breeds overall ranged between ΔCq values of 5–7, e.g., in tonsil (ΔCq 7), lung (ΔCq 5), ghLN (ΔCq 6), kidney (ΔCq 6), ingLN (ΔCq 5), and pLN (ΔCq 6), while differences in other organs were less pronounced, e.g., in mnLN (ΔCq 4), liver (ΔCq 4), and spleen (ΔCq 2). In contrast, Cq values in blood samples were lower for Pietrain boars, with a ΔCq of 3.

In gilts, the Cq values of the ASFV genomes from tissues tested did not differ considerably among the pens. Gilts that had to be euthanized due to reaching the humane endpoint (#534, #536, #627) or necropsied on day 9 pi per schedule (#539, #567) were highly positive by qPCR (Figure 4B). In these individuals, Cq values were mean 17.5 ± 2 in blood, 23.6 ± 5 in tonsil, 24.3 ± 4 in mnLN, 23.1 ± 1 in lung, 22.7 ± 4 in liver, 24 ± 2 in ghLN, 25.4 ± 2 in kidney, 20.6 ± 3 in spleen, 25.4 ± 2 in ingLN, and 24 ± 3 in pLN. All gilts euthanized according to schedule on day 34 and 35 pi showed comparable but slightly lower ASFV genome loads in organs, except blood (ΔCq 2.2), tonsil (ΔCq 3.4), mnLN (ΔCq 2.7), ingLN (ΔCq 3), and pLN (ΔCq 4.4). Notably, more gilts from pen 1 (#530–#576) reached the humane endpoint compared with pen 2 (#610–#654). However, all individuals except #610 in pen 2 contracted the virus later than individuals in pen 2, and scheduled necropsies were performed during starting/peak viraemia of these animals.

#### 3.3.2. Reproductive Organs

**Figure 5:**
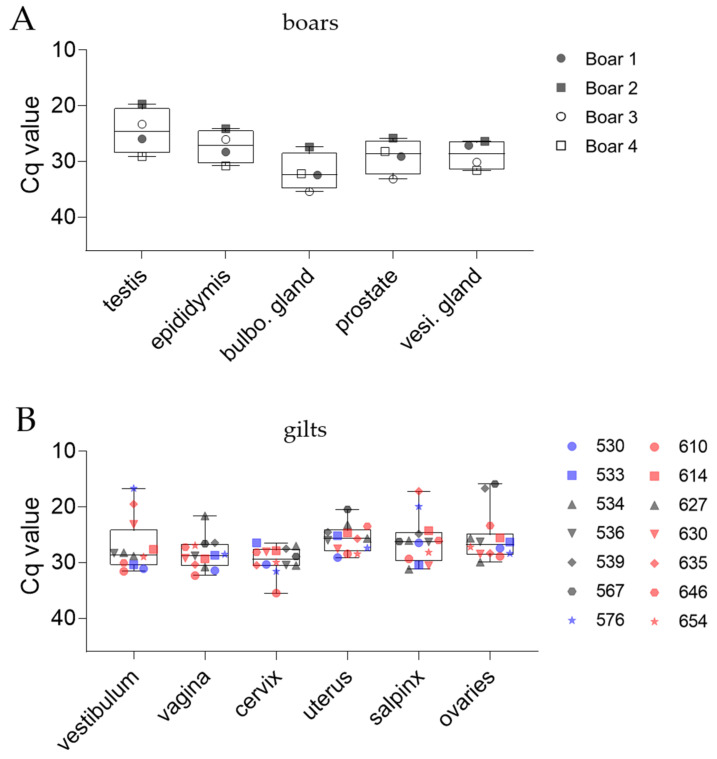
Detection of the ASFV genome in compartments of the porcine male (**A**) and female (**B**) reproductive tract by qPCR. The 25/75 percentiles are depicted as boxes with min. and max. values, while the group median is displayed as a line. Each individual is represented by a symbol. The grey color indicates animals that reached the humane endpoint before the scheduled trial end. Blue (pen 1) and red (pen 2) symbols represent individuals euthanized at the scheduled end of the trial.

Since data regarding the effects of ASFV infection on the reproductive organs of boars and gilts were rare, we evaluated the distribution of ASFV in all components of the porcine male and female reproductive tracts. Distinct from other tissues, no breed-dependent differences in Cq values were observed in boar reproductive tissues (Figure 5A). Although boars were euthanized on different days pi, no notable correlation between low Cq values and progressing dpi was present (not shown). The Cq values ranged from 20–29 (#2; #4) in testes, 24–31 (#2; #4) in epididymis, 27–35 (#2; #3) in bulbourethral glands, 26–33 (#2; #3) in prostates, and 26–32 (#2; #4) in vesicular glands.

The Cq values in female reproductive tracts did not differ between the pens, which is consistent with results obtained from other organs. Mean Cq values were 27 ± 4 in vestibuli, 28 ± 3 in vaginas, 29 ± 3 in cervices, 26 ± 3 in uteri, 26 ± 5 in salpinges, and 26 ± 4 in ovaries (Figure 5B). 

#### 3.3.3. Embryos and Fetuses

**Figure 6:**
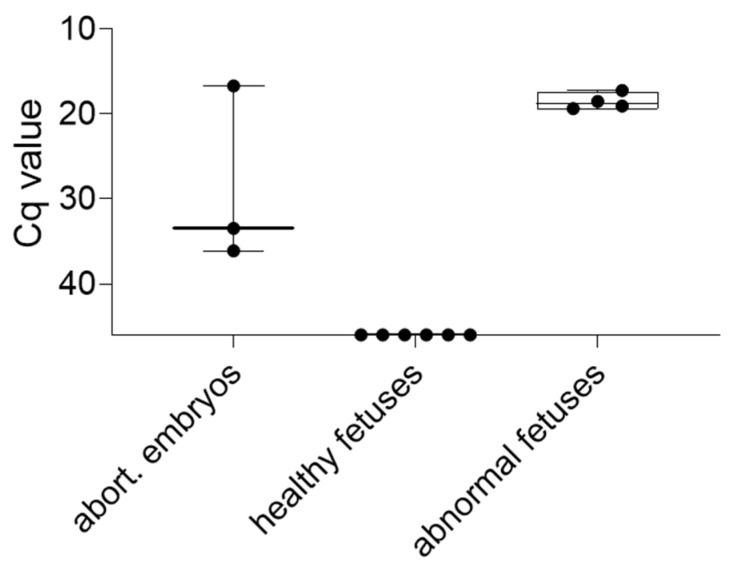
Detection of the ASFV genome in embryos and fetuses obtained during the trial. Specimens were divided into three groups based on macroscopic appearance. Boxes depict 25/75 percentiles with group median and min/max values. Each specimen is represented by a symbol.

Since 10/13 pregnant gilts aborted or resorbed implanted embryos upon the development of high fever (71.5% abortion rate), aborted embryos were tested by qPCR along with embryos and fetuses obtained during necropsies (Figure 6). Gilt #530 did not implant embryos after AI, based on the uterus and ovary morphology. Embryos/fetuses were divided into three groups: (I) aborted embryos, (II) healthy embryos/fetuses obtained during necropsy, (III) anomalous embryos/fetuses obtained during necropsy. Anomalous embryos/fetuses were deformed and/or presented with blood-shot amniotic fluid (originating from gilt #533, not shown). Strikingly, healthy appearing embryos/fetuses were negative by qPCR, while anomalous counterparts were highly positive, with mean Cq values of 19 ± 1. Aborted embryos/fetuses resulted in heterogenous Cq values of 33.4, 36, and 16.6. 

### 3.4. Assessment of Infectious Virus in Reproductive Organs, Semen and Spleen

To assess the impact of the ASFV infection on male and female reproductive tracts, HATs were performed on semen samples and samples of the male and female reproductive tracts. Spleen homogenates were included as a well-characterized reference (Table 1). 

All boar spleens were positive, except boar #3, which was euthanized after recovering from acute ASFV infection (25 dpi). All prostates were positive, but both Large White boars were highly positive compared with Pietrain boars. We observed comparable amounts of rousette formation in homogenates from vesicular glands. Furthermore, homogenates of the testis, epididymis, and bulbourethral glands of boar #3 were negative in HATs, while samples from all other boars were positive. Supporting the qPCR results, semen samples from boars #1 and #4 contained infectious ASFV at 2 dpi. Semen samples from day 0 pi were included as controls and remained negative in all pathogen detection tests. All semen samples were positive at day 3 pi, with exception of boar #2, which turned positive in semen at 4 dpi. Notably, Large White boars (#1 and #4) were positive at day 2 pi, while Pietrain boars (#2 and #3) were first positive at day 3 or 3 pi, respectively. 

Spleen samples of all gilts contained the infectious virus. Although gilts were euthanized on various days pi, nearly all reproductive tract homogenates were HAT-positive. Homogenates of the ovaries of gilts #536, #539, and #627 did not contain the infectious virus in any replicate tested.

### 3.5. Comparative Serology 

To assess seroconversion, all boar and gilt serum samples were tested using two commercially available ELISA kits. In the competition assay (Ingezim PPA COMPAC), boar #1 was positive on the day of necropsy with an S/P% of 68.8% (11 dpi), whereas boar #2 remained negative until necropsy (37.6% at 10 dpi, Table 2). Boar #3 gave a positive result at day 14 pi (57.4%) and remained positive until the day of necropsy (86.9%). Boar #4 was positive at the day of necropsy (80%). In general, results were comparable with the indirect assay (ID Screen^®^), with the exception that boar #2 was positive at 10 dpi (40.4%). Validation via IPT revealed that all samples at ≥ 10 dpi were positive, with exception of boar #2 (Table 2).

In gilts, samples from early necropsied animals #539 and #567 produced negative results in all assays tested (Table 3). Gilts euthanized during the trial (#534, #536, #627) showed some conflicting opposing results in different assays. Gilt #534 was positive in the indirect (54.5%) and competition assay (71.9%), while gilt #536 gave a positive result in the competition (56.5%), but a questionable result in the indirect assay (37.3%). Gilt #627, euthanized at 33 dpi, was positive in competition (69.2%), but questionable in indirect ELISA (33.4%). Ultimately, the IPT revealed that all samples were negative, with the exception of gilt #627. In the competition assay, all remaining gilts were negative until day 28 pi (Table 3). Here, only gilts that were positive in PCR at day 7 pi turned positive: #530 (54.5%), #533 (57.1%), #576 (61.7%), and #610 (50.2%). All gilts were positive at necropsy (35, 36 dpi). In the indirect assay, all gilts were negative until necropsy. The IPT confirmed that all samples were positive starting at day 28 pi. Generally, blood swab samples had less sensitivity versus serum samples for both ELISAs.

## 4. Discussion

Often referred to as the ’forgotten pandemic’ [24], ASF has now impacted pig populations in 35 countries with a total of almost 2 million registered losses of domestic pigs as of October 2022 [25]. Although our knowledge of ASFV ecology has grown over the past 15 years, the ongoing spread of ASFV highlights the need to define and fully elucidate all possible routes of transmission. 

The modern pork industry largely relies on AI to optimize productivity and allow for insemination of numerous sows at one time [26]. Since it is not possible to freeze boar semen for later use, quality control measurements must be implemented quickly to preserve the viability of spermatozoa. This industry requirement must be juxtaposed with the possibility of ASFV dissemination via the use of boar semen. The 1997–1998 classical swine fever virus (CSFV) outbreak in The Netherlands provides some idea of the possible scale of the problem [26]. That is, over 1000 sow farms across The Netherlands were closed after receiving semen potentially contaminated with CSFV from affected boar studs [27]. Similarly, purchased boar semen as a source of PRRSV infection in sows was documented in 2016 [28].

In this context, we evaluated the possibility of ASFV transmission via semen from infected boars and found an infection rate of 50% when using standard AI procedures and ASFV-contaminated semen. The size of the study (4 boars, 14 gilts, one ASFV strain) introduces the risk of overgeneralization of the data, but the results provided clear evidence that recipient females are readily infected with ASFV via AI. Likewise, daily monitoring of semen samples allowed insight into ASFV kinetics and effects on boar semen. The ASFV genome and infectious virus were detected in semen as early as day 2 pi after intramuscular inoculation. This is comparable to PRRSV kinetics, in which semen becomes PCR-positive at day 2 pi in some boars [29] and PCR and swine bioassay positive at day 3 pi (when boars were collected at day 1 and 3 dpi) [30].

Notably, in modern boar studs, quality control is conducted on each batch of semen and routinely checked for (I) erythrocytes in semen, (II) motility of spermatozoa, (III) deformations, and (IV) spermatozoa count ([31], BHZP). However, no change in spermatozoa morphology or motility was induced by daily sampling, and even acute ASFV viraemia had no detectable effect on spermatozoa motility or morphology. Since none were altered by early ASFV infection in any of the boars included in this study, routine testing for the ASFV genome in boar semen should be considered in risk assessment procedures. However, as early detection of ASFV infection even before the manifestation of clinical signs is key for effective disease control, it is important to note that high Cq values require replicates for accurate data interpretation [32]. Additionally, utilizing the cell fraction (vs. whole semen) may be useful to detect additional positive samples as shown in this study on 2 dpi (boar #3). The concentration of the semen cell fraction for PCR has been shown to increase detection of PRRSV in previous boar studies [30]. This could be due to a concentration of the virus-infected macrophages or density of free virus in the cell fraction [33], but further studies need to be performed with ASFV to describe its entry into semen. Regardless of how the virus enters semen, to ensure detection of low genome loads and therefore early detection of ASFV infection, a detection pipeline with high sensitivity and specificity needs to be defined.

As quick dispatch of semen samples is essential for optimal viability and offspring numbers, we assessed the suitability of other matrices for early detection of ASFV infection in boar studs, although on a limited scale. It was previously described that blood samples show the highest accuracy in ASFV genome detection [23]; however, especially larger boar studs favor non-invasive strategies. Thus, oral fluids and rectal samples were examined for suitability. We report that rectal swabs and fecal samples are inadequate matrices for early detection, as only a few samples rendered positive in qPCR, as described previously [34]. Oral fluid samples allowed early detection of the ASFV genome at day 2 pi (mean Cq 37.9 ± 0.05 SD), a result consistent with results on pen-based oral fluid samples obtained from larger pens of pigs at 3 dpi (mean Cq 38.2 [35]). Furthermore, oral samples contained higher amounts of the ASFV genome early after inoculation (up to 5 dpi) than fecal samples, further indicating the suitability of this matrix to detect ASFV infection early with a non-invasive strategy. Overall, semen and oral fluid samples contained low but comparable amounts of ASFV genome relative to EDTA blood. Upon consideration of the fact that collection of EDTA blood during dummy mounting did not result in any irritated behavior, EDTA blood remains the most suited matrix for early ASFV genome detection. In view of Cq values, while sampling of semen resulted in lower values than oral fluids, this matrix cannot be obtained and screened daily. Additionally, testing an adequate number of samples using assays of high sensitivity will be crucial for early detection and to prevent ASFV dissemination via boar semen.

In addition to the kinetics of ASFV genomes and infectious particles in boar semen, we have provided the first evidence that ASFV can successfully be transmitted from boars to gilts via AI. Importantly, no differences in the severity or progression of the disease could be observed between venereal infection and possible contraction from pen mates by ingestion of infectious fluids (blood, oral fluid). The possibility of age-dependent venereal ASFV transmission to gilts or sows remains to be investigated, but it can be hypothesized that the initial insemination of gilts might result in microlesions as the gilt reproductive tract and cervix are smaller compared with those of sows [36], thereby facilitating viral entry. Additionally, the mechanism underlying the heterogenous appearances and qPCR results of embryos/fetuses remains to be elucidated.

Previous studies indicated that ASFV did not enter live spermatozoa of spiked boar semen [13], but simultaneous entry of virus and spermatozoa into the ovum cannot be refuted at this point. Attachment of virions to molecules on spermatozoa has been formerly described for other DNA viruses, such as the human papilloma virus (HPV, [37]). Alternatively, heterogeneity possibly results from compartmentation of the porcine uterus, since migration of infected macrophages from circulation into the uterus is unlikely due to placental morphology. However, it has been shown that PRRSV transmission from pregnant sows to their fetuses is linked to target macrophages being present in the endometrium and placenta, enabling transmission to fetuses [38]. Histopathological investigation could offer insight into the localization of these target macrophages. Overall, ASFV can successfully be transmitted from infected boars to gilts via AI, and 71.5% of gilts included in this study aborted at the onset of fever. Abortion rates of this magnitude would significantly reduce breeding efficiency but is of lesser importance for risk assessment procedures because affected gilt/sow herds would be culled upon outbreak detection.

In terms of the timing and detectability of the virus in various organs and body fluids, including semen, intramuscular infection represented a worst-case scenario. Studies with other strains of ASFV showed that the incubation period following IM inoculation can be hours to days shorter and is much more efficient than the natural route of transmission, which is usually oro-nasal in the absence of the tick vector [39].

In the current study with a complex and interlinked design, the intramuscular scenario was implemented to ensure that the remainder of the study could be conducted. Thus, proof of concept was provided, but future studies should also target the natural route and its shedding kinetics.

## 5. Conclusions

With this study, we present definitive evidence that ASFV can successfully be transmitted from infected boars to recipient gilts via AI. Infectious virions were found in semen samples as early as day 2 post-IM inoculation, and semen samples remained positive by both qPCR and hemadsorption test for at least 20 days. Gilts that contracted the virus via AI still implanted embryos, but most aborted upon development of a high fever. Moreover, we demonstrated that a proportion of fetuses harbored replicating viruses.

These findings, in tandem with insemination procedures used in the modern pork industry, indicate that venereal transmission of ASFV should be considered in future risk assessment strategies. Furthermore, we present data on alternative, non-invasive strategies to enable early detection of ASFV in boar studs, albeit these approaches will require further investigations.

## Figures and Tables

**Table 1:** Summarized results of hemadsorption tests. The presence or absence of infectious virus was evaluated in male and female reproductive organs, as well as semen samples.

Matrix	Boars				Gilts													
	1	2	3	4	530	533	534	536	539	567	576	610	614	627	630	635	646	654
Spleen	••	•••	—	••	••	••	••	••	•••	••	••	•	••	••	••	••	•	••
Testis	••	•••	—	••														
Epididymis	••	••	—	••														
Bulbourethral gland	•	••	—	•														
Vesicular gland	••	••	••	••														
Prostate	•••	•••	••	••														
Semen 0 dpi	—	—	—	—														
Semen 2 dpi	••	—	—	••														
Semen 3 dpi	••	—	••	•														
Semen 4 dpi	•••	••	••	•••														
Semen 5 dpi	•••	•••	••															
Semen 14 dpi			••															
Semen 20 dpi			••															
Vagina					•	•••	••	••	•••	••	••	••	•••	••	••	••	•	••
Cervix					••	•••	•••	••	••	•••	••	•	••	••	•	••	••	••
Uterus					•	••	•••	•••	•••	••	•••	••	•••	•••	••	••	••	••
Salpinx					•••	•••	•••	•••	•••	•••	••	•••	••	••	••	••	••	••
Ovaries					•••	•••	•••	—	—	•••	••	•••	••	—	•	••	••	•••

Classification: — negative; • weak positive; •• positive; ••• strong positive, grey area = no sample.

**Table 2:** Comparative serology of serum samples of boars. All samples were subjected to an indirect and a competition ELISA; positive/questionable samples were validated in IPT.

	ID Screen^®^ ASF Indirect [S/P%]				INgezim PPA COMPAC [%]				IPT			
**Sample [dpi]/OM**	1	2	3	4	1	2	3	4	1	2	3	4
−3	—	—	—	—	—	—	—	—	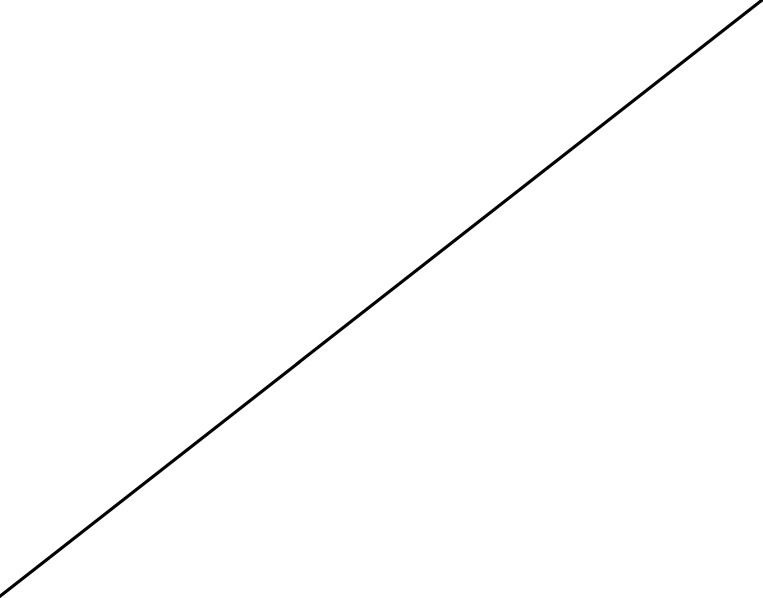			
−2	—	—	—	—	—	—	—	—				
−1	—	—	—	—	—	—	—	—				
0	—	—	—	—	—	—	—	—				
2	—	—	—	—	—	—	—	—				
3	—	—	—	—	—	—	—	—				
4	—	—	—	—	—	—	—	—				
5	—	—	—	—	—	—	—	—				
10		40.4				37.6				—		
*11*	49.9				68.8				+			
14			60.8				57.4				+	
*17*				72.7				80.0				+
20			48.6				79.9				+	
*25*			58.7				86.9				+	

Classification: — negative; grey area = no sample; / = not assessed in IPT.

**Table 3:** Comparative serology of blood swab/serum samples of gilts. All samples were subjected to an indirect and a competition ELISA, positive/questionable samples; as well, all serum samples obtained during necropsies were validated in IPT.

		Pen 1							Pen 2						
	**Sample [dpi]/OM**	530	533	534	536	539	567	576	610	614	627	630	635	646	654
**ID Screen^®^ ASF Indirect** **[S/P%]**	7	—	—	—	—	—	—	—	—	—	—	—	—	—	—
	9					11.1	24.7								
	14	—	—	—	—			—	—	—	—	—	—	—	—
	17				37.3										
	20			54.5											
	21	—	—					—	—	—	—	—	—	—	—
	28	14.9	17.5					19.3	13.4	—	—	—	—	—	—
	33										33.4				
	35	71.0	66.5					71.5		55,9				60.7	
	36								69.8			49.0	63.5		52.0
**INgezim PPA COMPAC** **[%]**	7	—	—	—	—	—	—	—	—	—	—	—	—	—	—
	9					18.9	14.4								
	14	—	—	—	—			—	—	—	—	—	—	—	—
	17				56.5										
	20			71.9											
	21	—	—					—	—	—	—	—	—	—	—
	28	54.5	57.1					61.7	50.2	—	—	—	—	—	—
	33										85.9				
	35	90.4	91.1					96.1		69.2				64.8	
	36								98.0			79.4	71.1		67.1
**IPT**	7														
	9					—	—								
	14														
	17				+										
	20			+											
	21														
	28	+	+					+	+						
	33										+				
	35	+	+					+		+				+	
	36								+			+	+		+

Classification: — negative; grey area = no sample; / = not assessed in IPT.

## Data Availability

Data are available on request from the corresponding author.

## Supplementary Materials

The following supporting information can be downloaded at: https://www.mdpi.com/article/10.3390/pathogens11121539/s1, Figure S1: Suitability of rectal swabs and feces for early ASFV detection.
