# Supplementary material for: Artificial Insemination as an Alternative Transmission Route for African Swine Fever Virus
Source: Pathogens. 2022 Dec 14;11(12):1539. doi: 10.3390/pathogens11121539 (PMC9785317; doi:10.3390/pathogens11121539)

Artificial insemination as alternative transmission route for African Swine Fever Virus
Supplementary Figure S1.: Viral genome in rectal swabs and feces of boars.

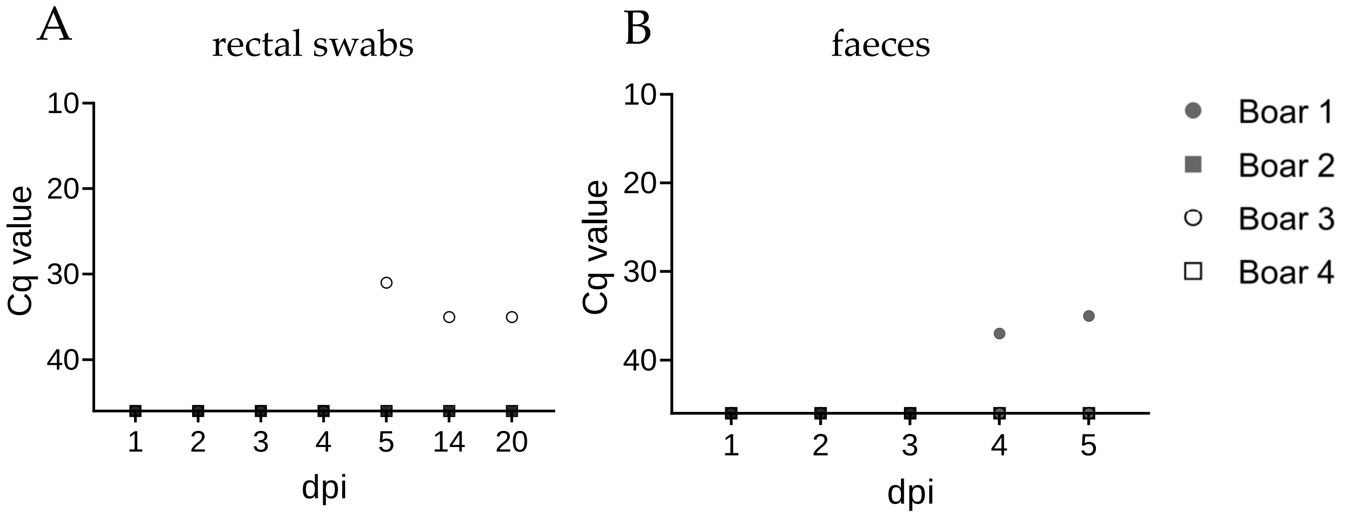

Supplement: Supplementary file 1 [file pathogens-11-01539-s001.zip › pathogens-2080421-supplementary.pdf]
